# An evolutionarily conserved phosphatidate phosphatase maintains lipid droplet number and endoplasmic reticulum morphology but not nuclear morphology

**DOI:** 10.1242/bio.028233

**Published:** 2017-09-27

**Authors:** Anoop Narayana Pillai, Sushmita Shukla, Abdur Rahaman

**Affiliations:** School of Biological Sciences, National Institute of Science Education and Research (NISER)-Bhubaneswar, HBNI, P.O. Jatni, Khurda 752050, Odisha, India

**Keywords:** Phosphatidic acid hydrolase, Lipin, *Tetrahymena thermophila*, Lipid droplet, Nuclear membrane expansion, Endoplasmic reticulum

## Abstract

Phosphatidic acid phosphatases are involved in the biosynthesis of phospholipids and triacylglycerol, and also act as transcriptional regulators. Studies to ascertain their role in lipid metabolism and membrane biogenesis are restricted to Opisthokonta and Archaeplastida. Here, we report the role of phosphatidate phosphatase (*PAH*) in *Tetrahymena thermophila*, belonging to the Alveolata clade. We identified two *PAH* homologs in *Tetrahymena*, *TtPAH1* and *TtPAH2*. Loss of function of TtPAH1 results in reduced lipid droplet number and an increase in endoplasmic reticulum (ER) content. It also results in more ER sheet structure as compared to wild-type *Tetrahymena*. Surprisingly, we did not observe a visible defect in the nuclear morphology of the Δ*Ttpah1* mutant. *TtPAH1* rescued all known defects in the yeast *pah1*Δ strain and is conserved functionally between *Tetrahymena* and yeast. The homologous gene derived from *Trypanosoma* also rescued the defects of the yeast *pah1*Δ strain. Our results indicate that *PAH*, previously known to be conserved among Opisthokonts, is also present in a set of distant lineages. Thus, a phosphatase cascade is evolutionarily conserved and is functionally interchangeable across eukaryotic lineages.

## Abstract (precis)

**Summary:** *Tetrahymena* possesses two PAH homologs, *TtPAH1* and *TtPAH2*. *TtPAH1* regulates lipid droplet biogenesis and ER morphology in *Tetrahymena* and functionally replaces yeast *PAH1.*

## INTRODUCTION

Eukaryotic cell organelles are enclosed by a membrane composed of the lipid bilayer and proteins. Phospholipids constitute the major structural components of lipid bilayers and play a central role in membrane biogenesis, lipid metabolism and signaling (Van Meer et al., 2008). The lipid composition of the membrane is critical for maintaining the shape, size and number of organelles, and is established through synthesis, transport and modification of phospholipids (McMahon and Gallop, 2005). The regulation of lipid synthesis and storage is critical for maintaining lipid homeostasis since both excess and poor fat storage results in various lipid-associated disorders (Klingenspor et al., 1999; Reue et al., 2000; Péterfy et al., 2001). However, the molecular mechanisms that link lipid production to organelle morphology remain unclear.

**Fig. 1.**
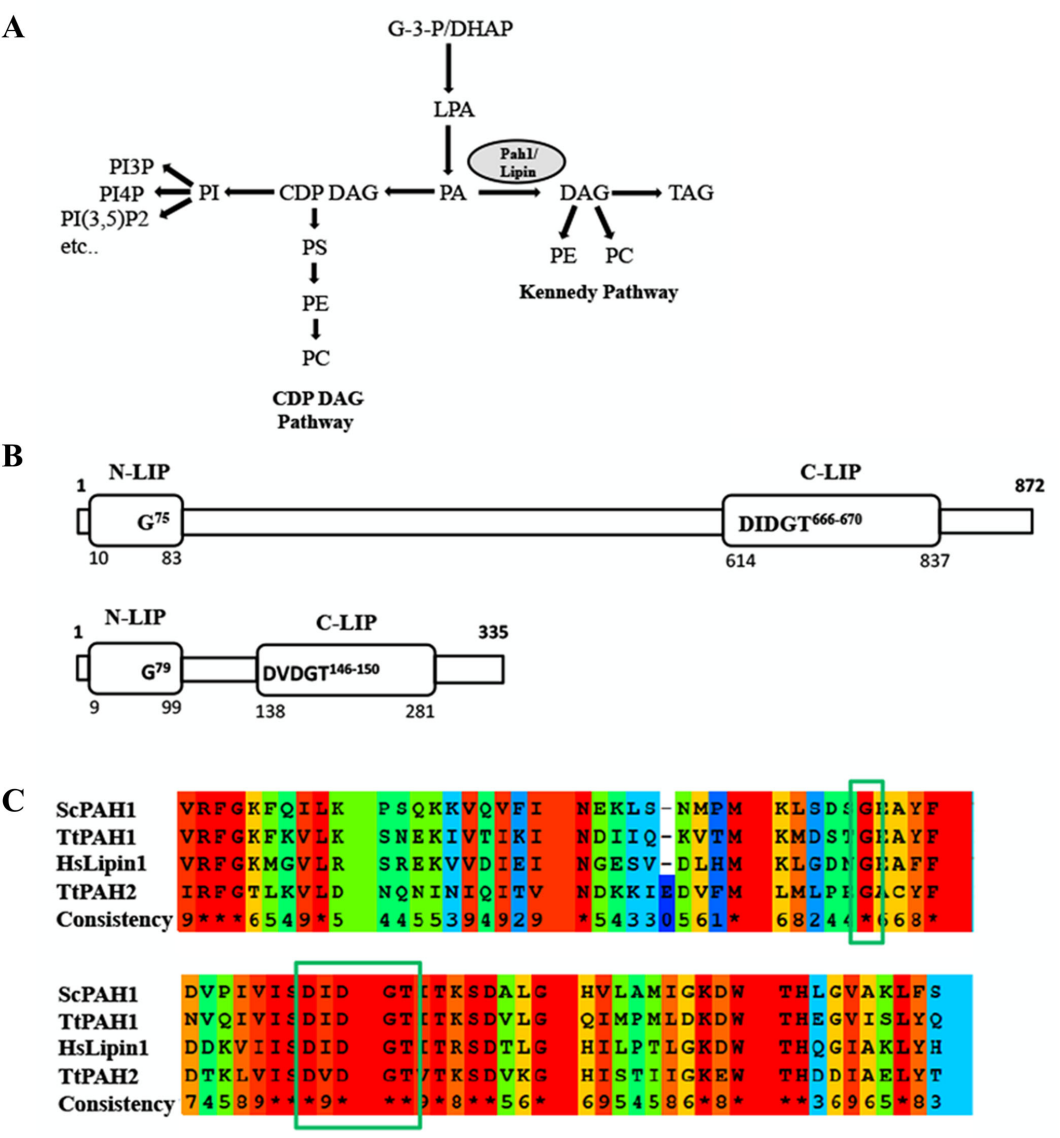
**Domain organization, sequence analysis and function of PAH protein.** (A) Schematic representation of the role of PAH in lipid biosynthesis. PA is a key precursor used for the synthesis of PE and PC through the CDP-DAG pathway. In the presence of choline and ethanolamine, these phospholipids are synthesized through the Kennedy pathway. In metazoans, the pathway that converts CDP-DAG to PC/PE (CDP-DAG pathway) does not exist, whereas both the pathways are present in yeast. G-3-P, glycerol-3-phosphate; LPA, lysophosphatidate; PI3P, phosphatidylinositol-3-phosphate; PI4P, phosphatidylinositol-4-phosphate; PI(3,5)P2, phosphatidylinositol-3, 5-biphosphate; PS, phosphatidylserine. (B) Domain organization of TtPAH1 and TtPAH2**.** Predicted N-LIP and C-LIP domains are indicated in the boxes. Also shown are the positions of a conserved glycine residue in N-LIP and the HAD with its conserved DXDXT/V motif in C-LIP. (C) Multiple sequence alignment showing partial sequences of N-LIP (top) and C-LIP (bottom) of PAH proteins from *T.* *thermophila*, *S.* *cerevisiae* and *H.* *sapiens*. Assigned colors of the particular residues are based on alignment consensus. Conserved glycine residue in N-LIP and catalytic motif (DXDGT/V) in C-LIP are indicated inside the box.

Pah/lipin proteins are Mg^2+^-dependent phosphatidic acid phosphatases (3-sn-phosphatidate phosphohydrolase, EC 3.1.3.4) (Han et al., 2006). Members of the Pah/lipin protein family perform dephosphorylation of phosphatidic acid (PA) to generate diacylglycerol (DAG), the penultimate step in glycerolipid synthesis (Lin and Carman, 1989). DAG can be converted back to PA by DAG kinase. PA and DAG are the central precursors which control the levels of phospholipids, govern membrane structure and lipid storage. In yeast, phospholipid biosynthesis occurs by two pathways: the cytidine diphosphate diacylglycerol (CDP-DAG) pathway (*de novo*) and the Kennedy pathway (salvage) (Fig. 1A) (Carman and Zeimetz, 1996; Carman and Henry, 1999). DAG is converted to triacylglycerol (TAG) (Han et al., 2006), which forms the lipid droplet. The dual function of TAG, as a reservoir of cellular energy and precursor for membrane phospholipids, makes it a key player in lipid homeostasis. DAG derived from PA is used for the synthesis of membrane phospholipids phosphatidylethanolamine (PE) and phosphatidylcholine (PC) via the Kennedy pathway (Carman and Kersting, 2004). Through the CDP-DAG pathway, phosphatidic acid serves as the precursor for the synthesis of the phospholipids PE, PC and phosphatidylserine (PS). Apart from the synthesis of lipids, PA and DAG act as lipid second messengers in signaling events that trigger membrane expansion, secretion and endocytosis (Kearns et al., 1997; Nadra et al., 2008). In yeast, PA positively regulates phospholipid synthesis through sequestration of a transcription repressor Opi1, thereby activating the transcription of genes encoding lipid biosynthetic enzymes (White et al., 1991; Loewen et al., 2004).

Lipins are relatively large proteins close to 100 kDa, and are primarily found in the cytosol. These proteins contain a carboxy-terminal region (C-LIP) with a haloacid dehalogenase (HAD)-like domain, possessing the DXDXT/V catalytic motif and an amino-terminal domain (N-LIP) of unknown function (Santos-Rosa et al., 2005). Lipin 1 was initially identified through positional cloning as the mutated gene in the fatty liver dystrophy (*fld*) mouse, which is characterized by abnormal development of adipose tissue that results in lipodystrophy and insulin resistance (Reue et al., 2000; Péterfy et al., 2001). In *Saccharomyces cerevisiae*, a single lipin orthologue, *PAH1*, is present, whereas mammals express three lipin paralogues, *LIPIN1*, *LIPIN2* and *LIPIN3*, exhibiting distinct but overlapping expression patterns (Han et al., 2006; Donkor et al., 2007). The first lipin protein shown to function as a Mg^2+^-dependent phosphatidic acid phosphatase enzyme was *S. cerevisiae* Pah1 (Han et al., 2006). Deletion of *PAH1* in yeast causes aberrant expansion of nuclear/endoplasmic reticulum (ER) membrane, increased phospholipid synthesis, decreased TAG level and lipid droplet number, and slow growth (Siniossoglou et al., 1998; Adeyo et al., 2011). In *Caenorhabdtis* *elegans*, downregulation of lipin affects the dynamics of the peripheral ER and nuclear envelope (Golden et al., 2009; Gorjánácz and Mattaj, 2009). Defects in mammalian lipins lead to various metabolic disorders including lipodystrophy and insulin resistance, rhabdomyolysis, peripheral neuropathy and inflammation (Reue et al., 2000; Müller-felber et al., 2010).

Besides serving enzymatic functions, lipins also act as transcriptional regulators (Finck et al., 2006; Zhang and Reue, 2017). Mammalian lipins regulate gene expression by modulating the activity of key transcription factors such as peroxisome proliferator-activated receptor γ (PPARγ), PPAR co-activator 1α (PGC-1α) and sterol regulatory element binding protein (Phan et al., 2004; Peterson et al., 2011; Kim et al., 2013). Yeast Pah1 translocates to the nucleus where it interacts with the promoter of phospholipid synthesis genes (Santos-Rosa et al., 2005).

Phosphorylation and dephosphorylation at multiple sites regulate the activity and subcellular localization of PAH proteins. In yeast, Cdc28 phosphorylation of Pah1 is critical for cell cycle progression while phosphorylation by Pho85 plays other roles; in mammals, mTOR kinases phosphorylate lipins (Laplante and Sabatini, 2009; Peterson et al., 2011; Choi et al., 2012). Dephosphorylation of Pah1 by a nuclear/ER membrane complex consisting of a catalytic phosphatase subunit nuclear envelope morphology protein 1 (Nem1), and its regulatory subunit, sporulation-specific protein 7 (Spo7), activates its catalytic function and recruits it to the ER membrane, where it acts on its substrate PA (Santos-Rosa et al., 2005; Karanasios et al., 2010).

Studies of phosphatidic acid phosphatase have focused on Opisthokonta (fungi, nematode, flies and mammals) (Santos-Rosa et al., 2005; Han et al., 2006; Donkor et al., 2007; Golden et al., 2009; Gorjánácz and Mattaj, 2009; Ugrankar et al., 2011) and Plantae (Nakamura et al., 2009) clades. These enzymes and the regulatory cascades in which they participate have not been reported in organisms including Amoebozoa, Alveolata and Excavata. *Tetrahymena thermophila* belongs to the Alveolata, a major evolutionary branch of eukaryotic protists, in which cells display functional complexity comparable to the cells of humans and other metazoans. In this study, we report the role of phosphatidic acid phosphatase (Pah) in regulating lipid homeostasis and membrane biogenesis in this ciliate. We also investigated the cellular functions of *PAH/LIPIN* homologs in Excavata to understand the evolutionary conservation of this cascade.

We found two homologs for *PAH* in the Tetrahymena Genome Database. The larger protein is TtPah1, and a smaller one is TtPah2. We investigated the role of *TtPAH1* in regulating lipid homeostasis, maintaining nuclear morphology and ER organization. We characterized the effects of loss of function of *TtPAH1* and also performed complementation studies in the *pah1*Δ yeast strain. Deletion of *TtPAH1* in *Tetrahymena* led to a reduction in lipid droplet number, thus confirming its role in lipid homeostasis. However, unlike in yeast, *TtPAH1* was not required to maintain nuclear morphology. Overall, we provide evidence for the evolutionary conservation of this Mg^2+^-dependent phosphatidic acid phosphatase in Alveolata and Excavata.

## RESULTS

### *Tetrahymena* harbors two *PAH* homologs

We identified two homologs of phosphatidic acid phosphatase (*PAH*/*LIPIN*) in the Tetrahymena Genome Database and designated them as *TtPAH1* (TTHERM_00189270) and *TtPAH2* (TTHERM_00215970). TtPah1 contains 872 amino acids and is comparable to Pah1 proteins in other organisms, whereas TtPah2 (335 amino acids) is smaller than other known lipins. Both TtPah1 and TtPah2 proteins possess two specific phosphatidic acid phosphatase (PAP) domains, N-LIP and C-LIP, suggesting that these are the Mg^2+^-dependent phosphatidate phosphatases (Fig. 1B). All Mg^2+^-dependent phosphatidic acid phosphatases contain an essential catalytic DXDXT/V motif in the HAD-like domain of the C-LIP region. This catalytic motif is present in the C-LIP domain of both TtPah1 (666 DIDGT 670) and TtPah2 (146 DVDGT 150) (Fig. 1B,C). While the amino acid sequence of TtPah1 has 24% identity with yeast Pah1 and 31% identity with human lipin, TtPah2 has 22% identity with yeast Pah1 and 34% with human lipin. Similar to other phosphatidic acid phosphatases, the amino acids are more conserved in the N-LIP (50% and 49% identity for TtPah1, 35% and 30% identity for TtPah2 with yeast Pah1 and human lipin1, respectively) and C-LIP regions (49% identity for TtPah1 and 44% identity for TtPah2 with both yeast Pah1 and human lipin1). A conserved G residue in N-LIP is critical for PAH function since its mutation in mammalian lipin1 causes lipodystrophy. We have also identified the conserved G residue in N-LIP of both TtPah1 (G75) and TtPah2 (G79) (Fig. 1B,C).

### TtPAH1 localizes on ER and encodes functional phosphatidate phosphatase

**Fig. 2.**
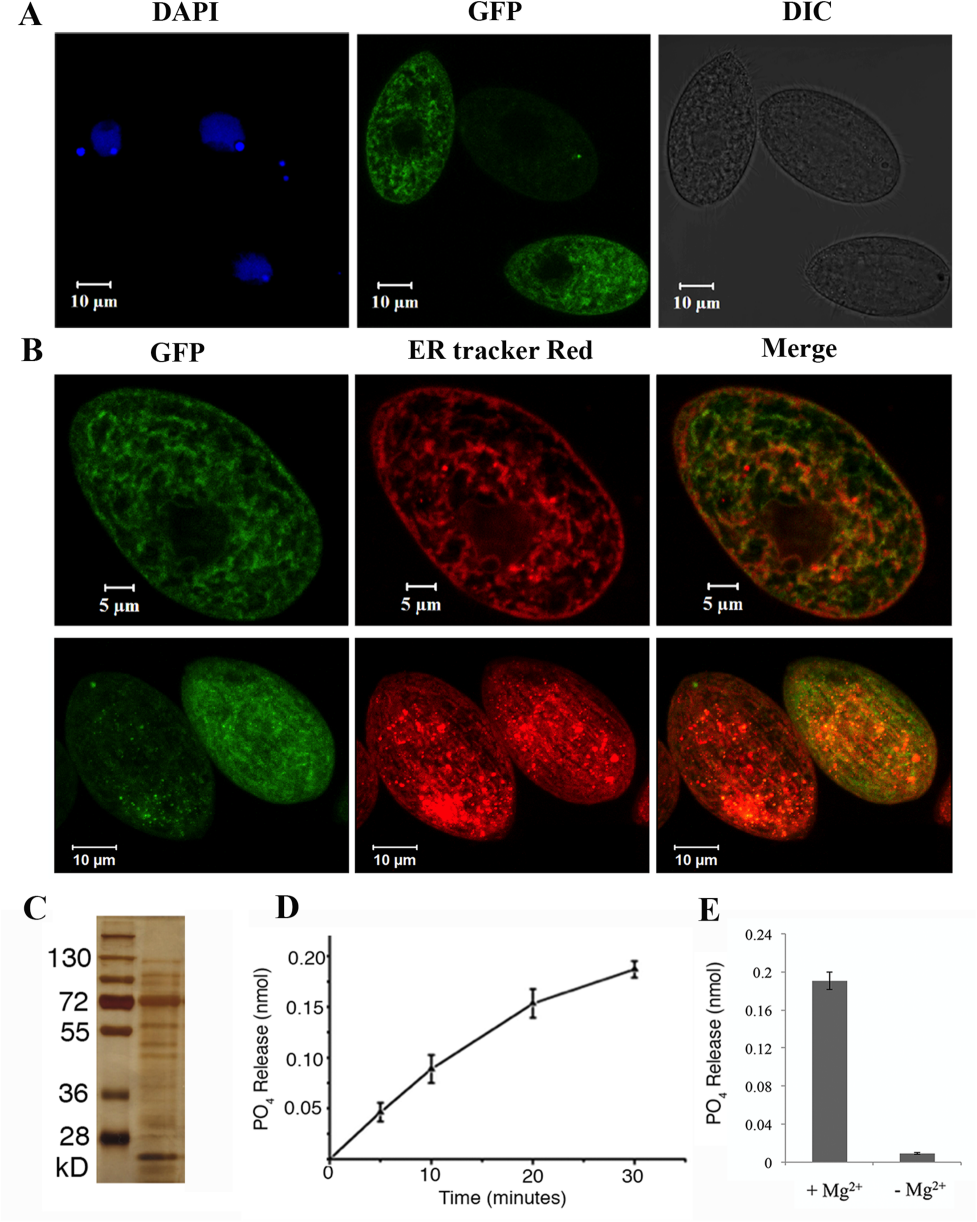
**TtPAH1 localizes on ER and encodes functional phosphatidate phosphatase.** (A) Localization of TtPAH1-GFP in *Tetrahymena* cells. Confocal image of fixed *Tetrahymena* cells expressing *TtPAH1-GFP* after DAPI staining; DAPI-stained nuclei (left), TtPAH1-GFP (middle) and DIC image of the fixed growing cell (right). (B) TtPAH1 associates with ER. Confocal images of fixed cells expressing *TtPAH1-GFP* after staining with ER-Tracker Red; TtPAH1-GFP (upper left), ER-Tracker Red (upper middle) and merge (upper right). Confocal stack of a different *Tetrahymena* cell expressing *TtPAH1-GFP* and stained with ER-Tracker Red is shown in the lower panels. (C) TtPAH1 purified as TAP-tag fusion in *Tetrahymena*. The silver-stained gel of purified TtPAH1 along with standard molecular weight marker is shown, with the molecular weights indicated on the left. In addition to the expected band of ∼100 kDa, many smaller species likely reflect partial proteolysis of the full-length protein. (D) TtPAH1 displays phosphatidate phosphatase activity. TtPAH1 protein (1 µM) purified from *Tetrahymena* was used to measure phosphatidic acid phosphatase activity, using a colorimetric assay. The average phosphate released (nmol) (*n*=3) was plotted against time. (E) The phosphatidate phosphatase assay performed either in the presence (+Mg^2+^) or absence (−Mg^2+^) of magnesium. The assay was carried out for 30 min before measuring the activity. TtPAH1 showed activity only in the presence of Mg^2+^, confirming it to be a PAP1 enzyme. The average phosphate released (nmol) (*n*=3) is shown.

We focused our study on *TtPAH1*. To assess its localization, we overexpressed it bearing a green fluorescent protein (GFP) tag. Analysis of confocal images showed that TtPah1-GFP was distributed throughout the cell (Fig. 2A). To evaluate whether TtPah1 associates with ER membrane, *Tetrahymena* cells expressing TtPah1-GFP were labeled with ER-Tracker Red dye, and analyzed by confocal microscopy. The results revealed that TtPah1-GFP is localized to ER membrane in addition to the cytoplasm (Fig. 2B). To examine whether *TtPAH1* encodes a functional phosphatidate phosphatase, we expressed a tandem affinity purification (TAP)-tagged fusion protein in *Tetrahymena.* We then purified the protein from lysates and measured phosphatidate phosphatase activity using a colorimetric assay. The purified protein migrated by sodium dodecyl sulfate polyacrylamide gel electrophoresis (SDS-PAGE) at its expected size, near 100 kDa, but there were also more abundant smaller species, probably corresponding to proteolytic products (Fig. 2C). This purified protein dephosphorylated PA in a Mg^2+^-dependent manner (Fig. 2D,E). Taken together, these results confirm that TtPah1 is a functional PAH in *Tetrahymena*.

### *TtPAH1* is dispensable for normal growth of *Tetrahymena* and loss of *TtPAH1* does not affect expression of *TtPAH2*

**Fig. 3.**
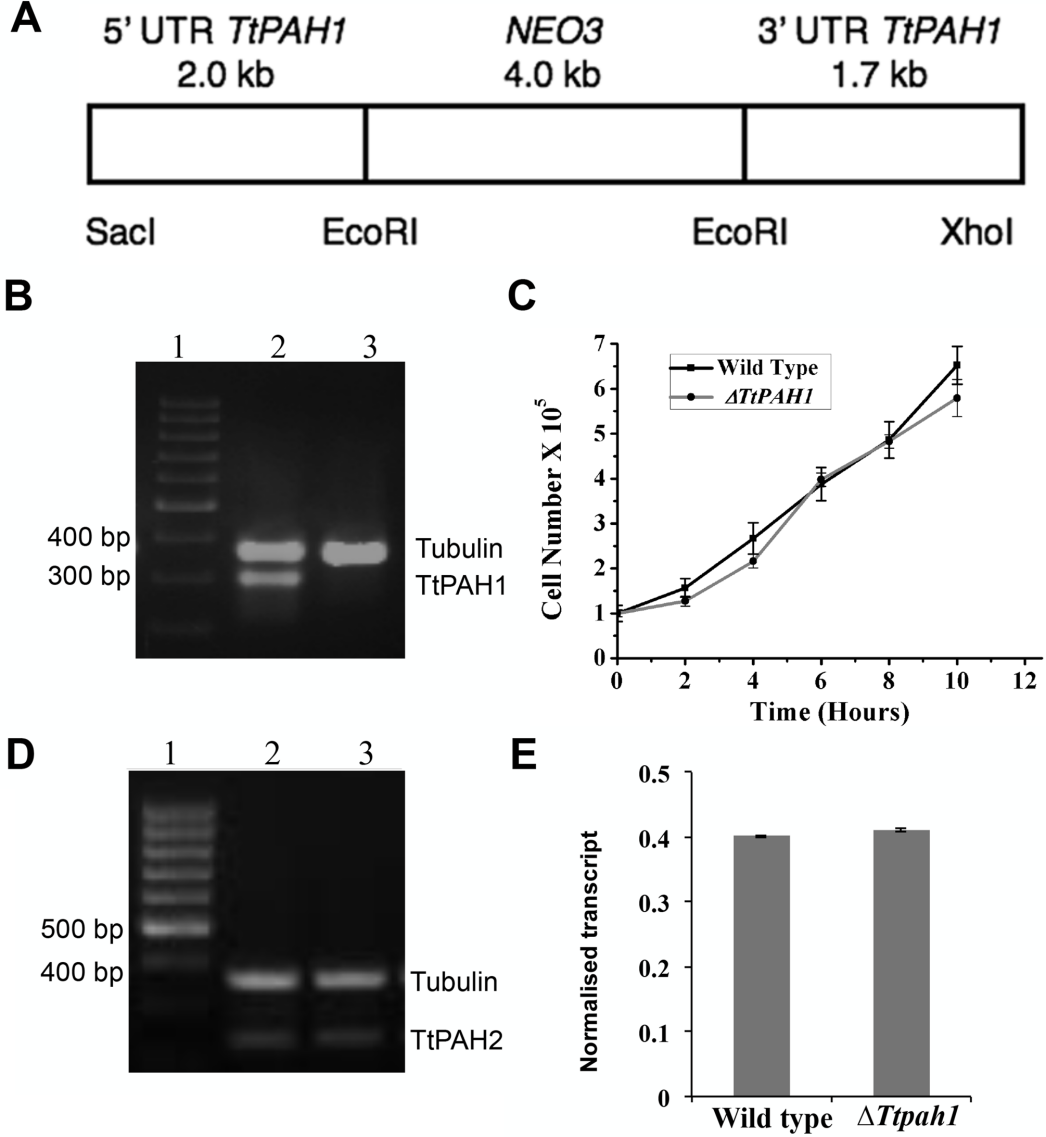
***TtPAH1* is dispensable for normal growth of vegetative *Tetrahymena* cells.** (A) Schematic showing organization of the knockout construct used to disrupt *TtPAH1* in the macronucleus. Gene disruption was performed by replacing the *TtPAH1* ORF with *NEO3* gene cassette, by homologous recombination. The *NEO3* cassette confers resistance to paromomycin. (B) RT-PCR analysis of wild-type and Δ*Ttpah1* cells. Lane 1, standard molecular weight marker; lane 2, amplified products of cDNA from wild-type cells; lane 3, amplified products of cDNA from Δ*Ttpah1* cells. The top band just below the 400 bp marker corresponds to alpha-tubulin (387 bp), and the band near 300 bp represents *TtPAH1*. The absence of a 300 bp band corresponding to *TtPAH1* confirms that knockout is complete. (C) Growth curve of *Tetrahymena* wild-type versus Δ*Ttpah1* cells. The cell numbers were counted every 2 h, and the number of cells/ml was plotted against time. Loss of *TtPAH1* does not affect *Tetrahymena* growth significantly. (D) Semi-quantitative RT-PCR showing expression of *TtPAH2* in wild-type and Δ*Ttpah1* cells. Lane 1, standard molecular weight marker; lane 2, amplified products of cDNA from wild-type cells; lane 3, amplified products of cDNA from Δ*Ttpah1* cells. The top band in lanes 2 and 3 corresponds to alpha-tubulin (387 bp), and the band near 238 bp represents *TtPAH2*. (E) The graph shows quantitation of *TtPAH2* after normalization with the alpha-tubulin band. The expression of *TtPAH2* is not enhanced by the loss of TtPAH1*.*

In many organisms such as *Saccharomyces cerevisiae*, *C. elegans* and *Drosophila melanogaster*, *PAH* is required for normal growth (Santos-Rosa et al., 2005; Golden et al., 2009; Ugrankar et al., 2011). To assess whether *TtPAH1* is essential for normal growth of *Tetrahymena*, we generated the knockout strain by removing all 45 copies of *TtPAH1* from the macronucleus of wild-type *Tetrahymena* by homologous recombination. The knockout strains thus generated (Δ*Ttpah1*) were analyzed by semi-quantitative reverse transcription polymerase chain reaction (RT-PCR), which confirmed the absence of *TtPAH1* transcripts (Fig. 3A,B). The growth of Δ*Ttpah1* cells was not significantly different from that of wild-type cells (Fig. 3C). Moreover, there was no visible defect in the morphology of the knockout cells (data not shown)*.* To rule out the possibility that the lack of growth defect in Δ*Ttpah1* is due to compensatory overexpression of *TtPAH2* in these cells, we compared the expression of *TtPAH2* in Δ*Ttpah1* with that in wild-type cells. The expression of *TtPAH2* was not enhanced in Δ*Ttpah1* cells (Fig. 3D,E)*.* Taken together, these results suggest that *TtPAH1* is dispensable for normal growth of *Tetrahymena.*

### *TtPAH1* is required to maintain lipid droplet number in *Tetrahymena*

**Fig. 4.**
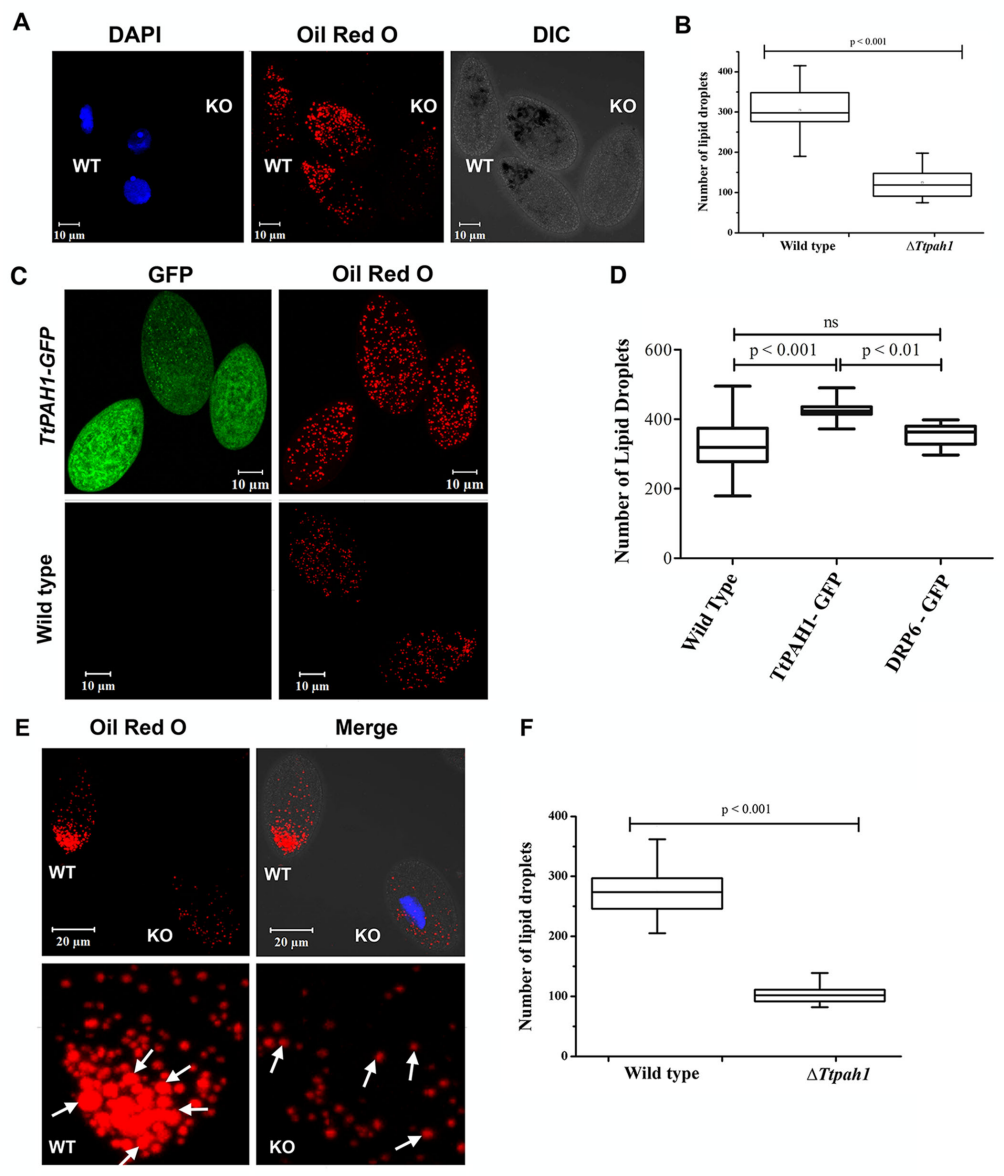
***TtPAH1* maintains lipid droplet number in *Tetrahymena*.** (A) Confocal images of *Tetrahymena* cells showing lipid droplets stained with Oil Red O dye. Wild-type cells and knockout cells were imaged together simultaneously. The wild-type cells were stained with DAPI to distinguish them from knockout cells. (B) Box plot showing the distribution of lipid droplet numbers in wild-type (*n*=35) versus Δ*Ttpah1* (*n*=38) cells. (C) Confocal images of wild-type and *TtPAH1-GFP*-expressing cells showing lipid droplets after staining with Oil Red O dye. (D) Box plot showing lipid droplet numbers in wild-type cells, cells overexpressing *TtPAH1-GFP* (*n*=20) and cells overexpressing *GFP-DRP6* (*n*=20). An increase in lipid droplet number is observed in cells expressing *TtPAH1-GFP*. (E) Confocal images of *Tetrahymena* cells showing lipid droplets stained with Oil Red O dye. Wild-type (WT) and knockout cells after starvation were imaged together simultaneously. Knockout cells were stained with DAPI to distinguish them from wild-type cells. Both the size and number of lipid droplets are reduced in Δ*Ttpah1* cells (KO). Lipid droplet size in wild-type cells appears to be larger than in the knockout cells as indicated by arrows. (F) Box plot showing lipid droplet numbers in wild-type (*n*=22) and Δ*Ttpah1* (*n*=22) cells under starved condition.

Lipid droplets are ubiquitous eukaryotic organelles mainly used for storing lipids (Murphy, 2001). They consist of a hydrophobic core of neutral lipids, such as triacylglycerol, sterols and sterol esters, surrounded by a phospholipid monolayer originating from the ER (Tauchi-Sato et al., 2002; Farese and Walther, 2009; Radulovic et al., 2013). Lipid droplet growth occurs either by localized synthesis of lipids or by fusion with other lipid droplets (Thiele and Spandl, 2008). Since Pah proteins are required for the synthesis of triacylglycerol, we compared lipid droplet numbers between Δ*Ttpah1* and wild-type cells. Lipid droplets were visualized by staining with Oil Red O, and the number of lipid droplets was counted after analyzing confocal images by LSM Image analyzer. The number of lipid droplets decreased significantly in Δ*Ttpah1* (Fig. 4A,B). Although there was no visible difference in the size of lipid droplets, quantitative analysis showed ∼60% reduction in lipid droplet numbers compared to wild type (Fig. 4B)*.* To provide further evidence that *TtPAH1* is involved in lipid droplet biogenesis, we overexpressed *TtPAH1-GFP* in wild-type *Tetrahymena* cells. Overexpression of *TtPAH1* resulted in a ∼20% increase in lipid droplet number compared to wild type (Fig. 4C,D). To demonstrate the specificity of this effect, we similarly overexpressed *DRP6-GFP* (a dynamin-related protein in *Tetrahymena*) and observed that it did not affect the lipid droplet number (Fig. 4D). Hence, we conclude that *TtPAH1* is required to maintain normal lipid droplet number in *Tetrahymena.* Decreased lipid droplet accumulation in Δ*Ttpah1* was not due to decreased nutrient uptake since we saw a similar reduction when the comparison between Δ*Ttpah1*and wild-type was performed under starvation conditions (Fig. 4E,F). Under starvation conditions, we observed a ∼60% reduction in lipid droplet number in Δ*Ttpah1* cells. Moreover, the size of lipid droplet in Δ*Ttpah1* was smaller than in wild-type cells (Fig. 4E). Taken together, these results suggest that *TtPAH1* influences the number and size of the lipid droplets in *Tetrahymena*.

### *TtPAH1* is needed for maintaining tubular ER in *Tetrahymena*

**Fig. 5.**
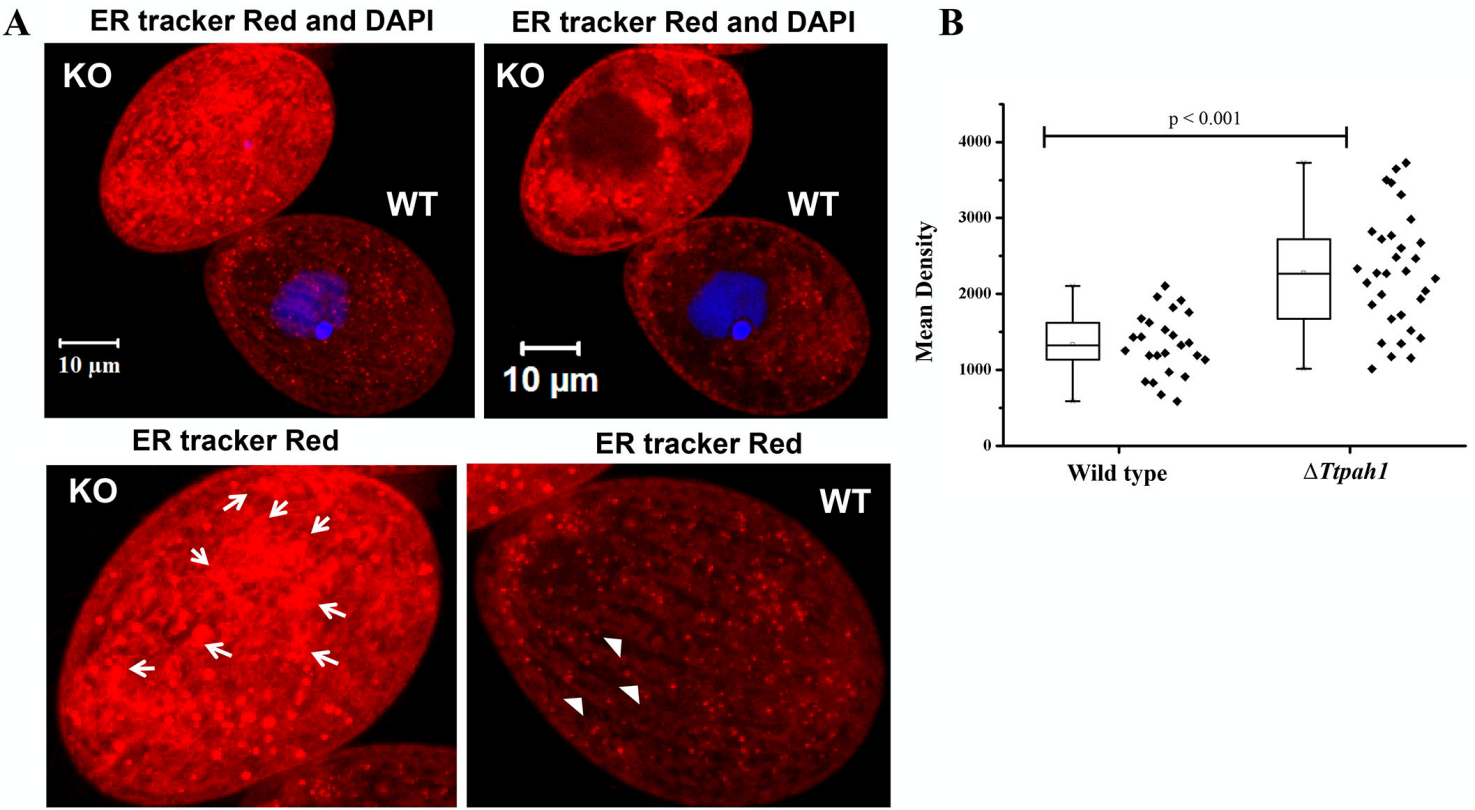
***TtPAH1* is needed for maintaining tubular ER.** (A) The top panel shows wild-type (WT) and Δ*Ttpah1* (KO) cells imaged simultaneously in the same field after staining with ER-Tracker Red. The left panel represents the confocal stack; the right panel is a single mid plane confocal slice. Wild-type cells were stained with DAPI to distinguish them from Δ*Ttpah1* cells. The enlarged images of Δ*Ttpah1* (bottom left) and wild-type (bottom right) cells are shown, indicating ER sheet (arrows) and ER tubule (arrowheads) structures. To rule out the effect of DAPI staining on ER morphology, we also stained Δ*Ttpah1* cells with DAPI and imaged them simultaneously with wild-type cells and found similar results*.* (B) Box plot showing the mean density of ER-Tracker Red staining. The mean intensity of Δ*Ttpah1* (*n*=32) is significantly higher than that of wild type (*n*=25).

The ER is a complex network consisting of flat sheets and highly curved tubules, and their abundance varies with cell cycle stages. The ER serves as the primary site for *de novo* lipid biosynthesis. We hypothesized that *PAH* regulates ER morphology since phosphatidic acid, a major component of ER, is converted to DAG by PAH. To determine whether *TtPAH1* is important in maintaining ER morphology, we stained both Δ*Ttpah1* and wild-type cells with ER-Tracker Red dye and analyzed morphology by confocal microscopy (Fig. 5A,B; Fig. S1). The ER content increased significantly in cells lacking *TtPAH1*, as measured by the mean density of ER-Tracker Red staining (Fig. 5C). Moreover, in wild-type cells, the ER appeared mainly as a network of fine tubules with occasional small patches, likely to represent ER sheets (Fig. 5A,B; Fig, S1). These patches seemed larger and more abundant in the absence of functional *TtPAH1*. This result suggests that *TtPAH1* is required for creating and/or maintaining the ER structure.

### Loss of *TtPAH1* does not manifest visible nuclear envelope defect in *Tetrahymena*

**Fig. 6.**
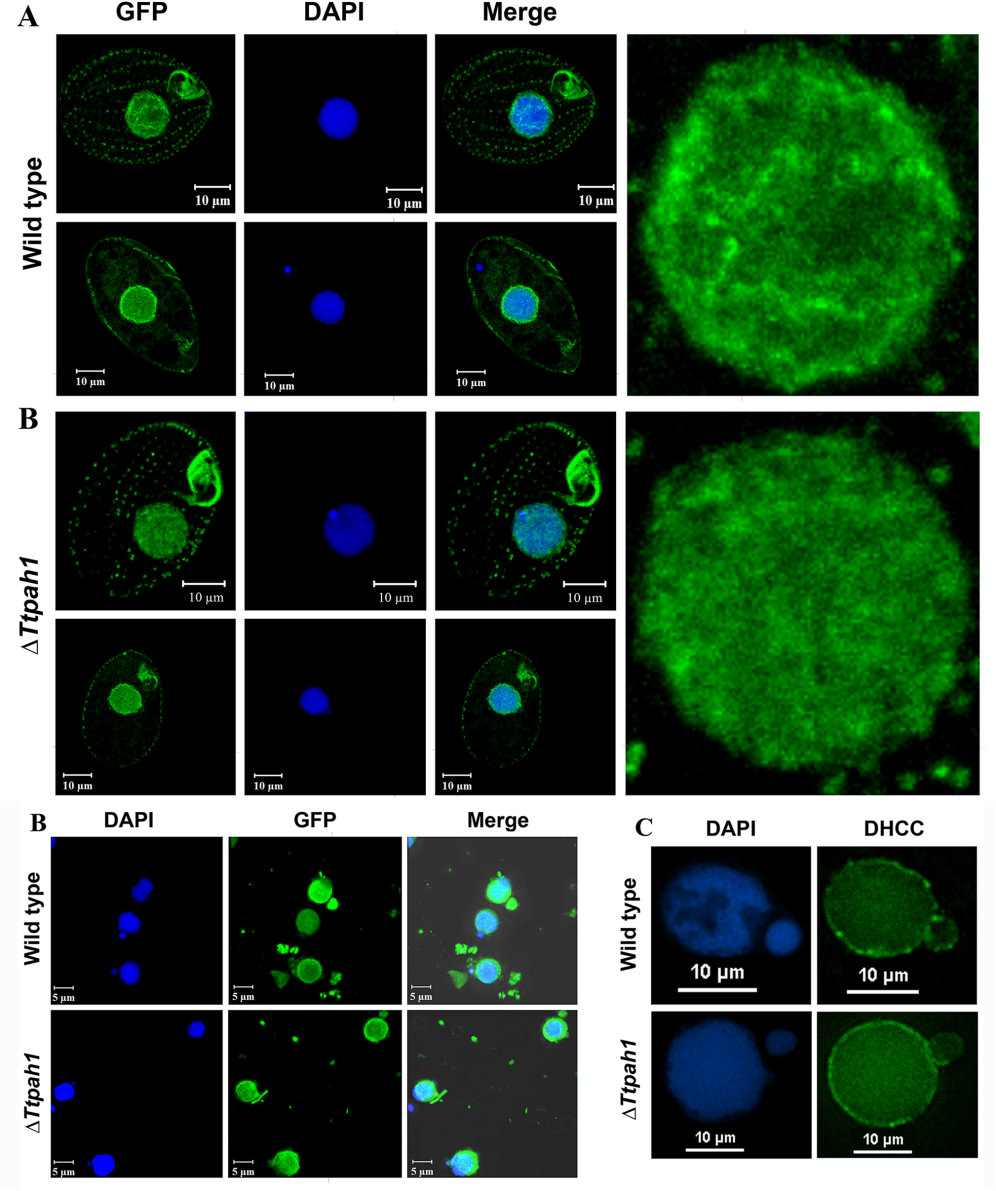
**Loss of *TtPAH1* does not manifest visible nuclear envelope defect in *Tetrahymena.*** (A) Confocal images of wild-type and Δ*Ttpah1* cells expressing *NUP3-GFP* after DAPI staining. In both wild-type and Δ*Ttpah1*, the upper panel is the Z-stack and the lower panel is a single slice. The enlarged nucleus from the Z-stack is shown on the right side. (B) Confocal images of DAPI-stained nuclei isolated from wild-type (upper panel) and Δ*Ttpah1* (lower panel) cells expressing *NUP3-GFP*. (C) Fluorescence images of *Tetrahymena* nuclei of wild type (upper panel) and Δ*Ttpah1* (lower panel) after staining with DHCC and DAPI. The images are deconvoluted using NIS Advanced Research software.

*Tetrahymena* harbors one polyploid, phenotypically active macronucleus (MAC) and a diploid transcriptionally silent germline micronucleus (MIC). To determine whether *TtPAH1* function is necessary to maintain normal nuclear envelope (NE) morphology, we analyzed the NE by expressing and visualizing *NUP3-GFP* (a nuclear pore component marker specifically localizing to macronucleus) in Δ*Ttpah1* cells and wild-type cells. This comparison did not reveal any visible defect in size or shape of the NE in Δ*Ttpah1* cells (Fig. 6A). Like in wild-type, the 4′,6-diamidino-2-phenylindole, dihydrochloride (DAPI)-stained DNA appeared round, compact and nonfragmented (Fig. 6A). Consistent with this, isolated DAPI-stained nuclei from wild-type and mutant cells expressing *NUP3-GFP* seemed identical (Fig. 6B). To further confirm that deletion of TtPAH1 did not affect nuclear morphology, we stained isolated nuclei (both MAC and MIC) with a lipophilic dye (3,3′-dihexyloxacarbocyanine iodide, DHCC) to visualize nuclear membrane. As with Nup3-GFP, we did not observe any visible defect in nuclear membranes of MAC (Fig. 6C). We also did not observe any detectable change in MIC structure (Fig. 6C). These results suggest that *TtPAH1* is not essential for maintaining normal nuclear morphology in *Tetrahymena.* Our results are in contrast to findings in *S. cerevisiae*, where cells lacking *PAH1* showed abnormal expansion of nuclear envelope that appeared as a nuclear membrane projections lacking DNA. Our results, taken together with our analysis of the ER, suggest that defects in ER morphology in *Tetrahymena* do not necessarily affect nuclear morphology, unlike the coupling in other organisms.

### *TtPAH1* restores different phenotypes of *pah1*Δ yeast cells

Though *TtPAH1* is not required for regulating nuclear expansion and nuclear shape in *Tetrahymena*, we asked whether the ciliate protein could rescue the nuclear defects in *S. cerevisiae pah1*Δ, which might be expected if the homologous proteins retain the same enzymatic activity. To assess nuclear morphology in budding yeast, we expressed nucleoplasmic protein PUS as a GFP-fusion and visualized *pah1*Δ cells expressing *TtPAH1*.

**Fig. 7.**
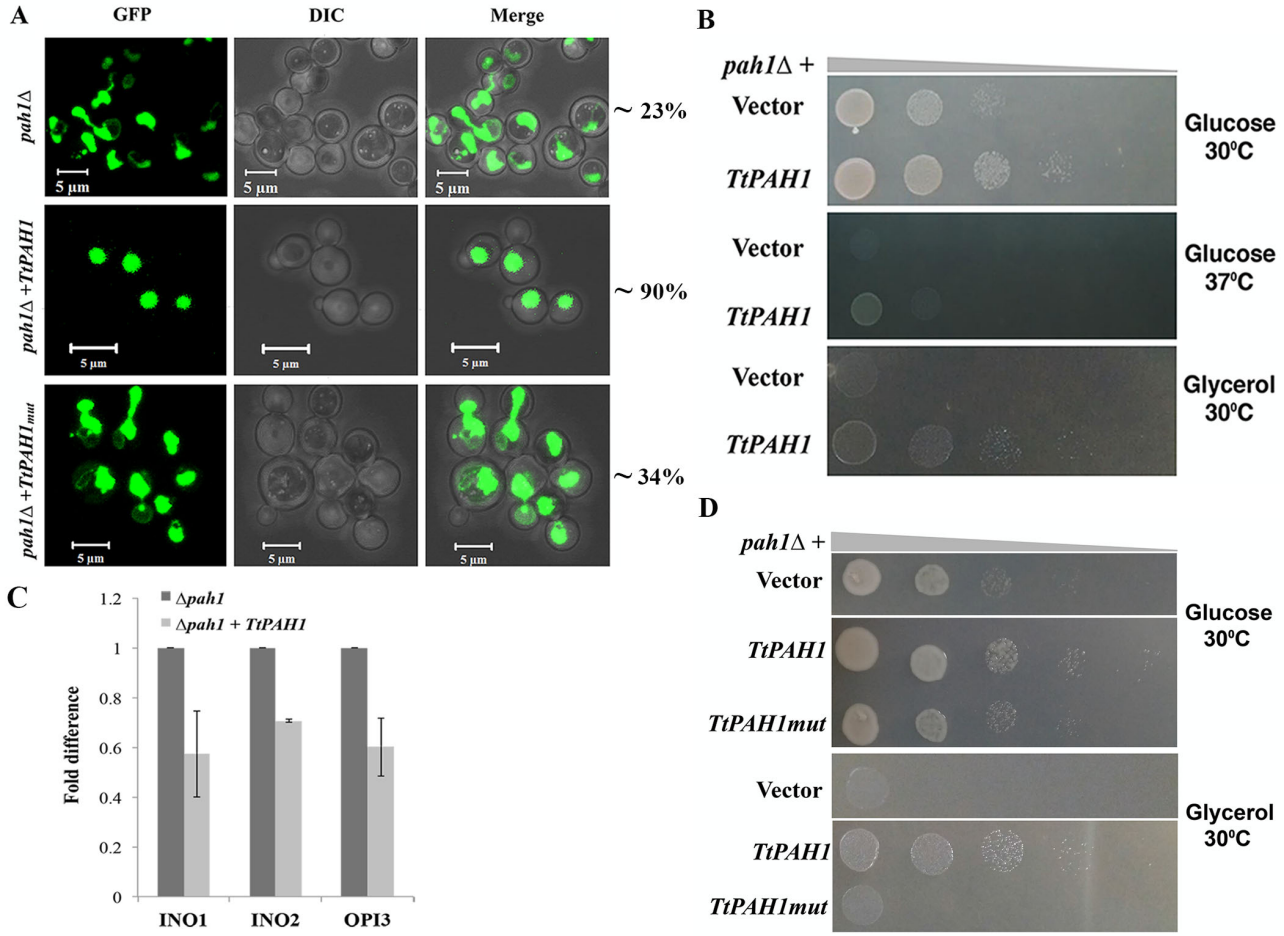
***TtPAH1* rescues the nuclear structure defect, slow growth phenotype and respiratory deficiency of *pah1*Δ yeast strain, and the catalytic motif is essential for its function.** (A) Confocal images of *pah1*Δ yeast cells transformed with either empty vector (top), *TtPAH1* (middle) or *TtPAHmut* (bottom) along with *PUS1-GFP* (an intranuclear reporter). *TtPAH1* but not *TtPAHmut* restores aberrant nuclei of *pah1*Δ yeast to wild-type spherical shape. Three different transformants per strain were analyzed and the number of cells counted for each transformant was 200-250 (*n*= 600-750). The percentage of cells containing round nucleus is indicated on the right. (B) The growth of *pah1*Δ yeast cells transformed with either *TtPAH1* or empty vector grown on SD media containing either glucose or glycerol (lacking leucine and uracil) at either 30°C or 37°C as indicated. The experiment was repeated three times. (C) Quantitative RT-PCR analysis of *INO1*, *INO2* and *OPI3* mRNAs in *pah1*Δ yeast cells transformed with either empty vector or *TtPAH1*. Amplification of each sample was performed in triplicate and normalized to a control gene *SEC63* in three independent experiments. (D) The growth of *pah1*Δ yeast cells transformed with either with empty vector, *TtPAH1* or *TtPAHmut* grown on SD media lacking leucine and uracil and containing either glucose or glycerol as indicated.

In *pah1*Δ, the nuclei in nondividing cells often appeared as two lobes interconnected by a long nuclear membrane extension (Fig. 7A) (Santos-Rosa et al., 2005). In contrast, *pah1*Δ expressing *TtPAH1* showed nearly normal nuclear morphology (Fig. 7A). This result suggests that *TtPAH1* can substitute for one or more functions of the yeast homolog.

*pah1*Δ also exhibits slow growth at 30°C, temperature-sensitive growth at 37°C (Han et al., 2006) and respiratory deficiency (i.e. growth defect) on nonfermentable carbon sources (Han et al., 2007). Along with rescue of the nuclear morphology defect, expression of *TtPAH1* restored growth both at 30°C and 37°C (Fig. 7B). To evaluate the role of *TtPAH1* in rescuing respiratory deficiency, we grew cells on plates containing glycerol as nonfermentable carbon source. The *pah1*Δ expressing *TtPAH1* grew faster than control *pah1*Δ cells (Fig. 7B).

Nuclear expansion in yeast is linked to the induction of phospholipid biosynthetic genes (Santos-Rosa et al., 2005). Deletion of *PAH1* induces the expression of inositol-3-phosphate synthase (*INO1*), the transcription factor *INO2* and phosphatidyl-N-methylethanolamine N-methyltransferase (*OPI3*), which are involved in the induction of phospholipid biosynthetic genes, leading to overly developed ER and aberrant expansion of nuclear membrane (Santos-Rosa et al., 2005). To test whether *TtPAH1* inhibits abnormal nuclear expansion in *pah1*Δ yeast by inhibiting the phospholipid biosynthesis genes, we have analyzed the mRNA levels of *INO1*, *OPI3* and *INO2* by quantitative real-time PCR using Sec 63 (a resident ER membrane protein unaffected by *PAH1* deletion) as a control (Santos-Rosa et al., 2005). *TtPAH1* repressed expression of all three genes tested, suggesting that *TtPAH1* could replace yeast *PAH1* in regulating expression of phospholipid biosynthesis genes (Fig. 7C). Taken together, these results suggest that *TtPAH1* retains all the known functions of yeast *PAH1*, and hence is functionally conserved between yeast and *Tetrahymena.*

A conserved DXDXT/V motif at C-LIP is essential for the catalytic activity of Pah1/lipin in yeast and mammals (Finck et al., 2006; Han et al., 2007). We identified a similar motif (666 DIDGT 670) in the predicted C-LIP of TtPah1 and evaluated if the motif is important for the function of *TtPAH1* by mutating two aspartate residues (D666,668E) (*TtPAH1_mut_*). Since *TtPAH1* functionally replaces yeast *PAH1*, we attempted to complement *pah1*Δ yeast cells with *TtPAH1_mut_*, and evaluated nuclear morphology, and growth in different temperatures and media. The mutant protein did not rescue aberrant nuclear morphology, slow growth at 30°C and the respiratory defect to the wild-type level (Fig. 7A,D). These results suggest that the catalytic activity of TtPah1 is important for its function.

### Phosphatidate phosphatase is conserved across eukaryotic lineages

**Fig. 8.**
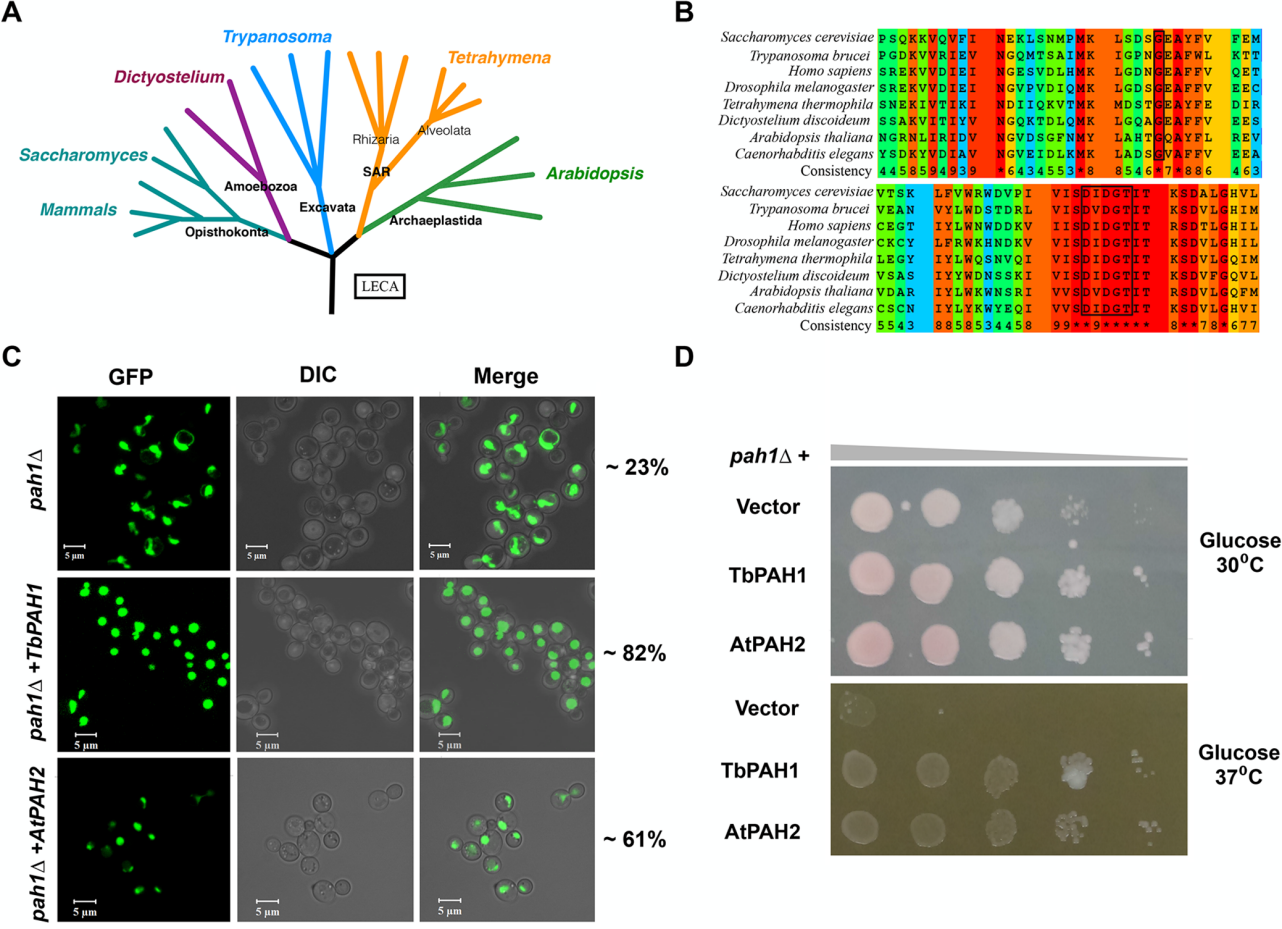
**Phosphatidate phosphatase is conserved across eukaryotic lineages.** (A) Eukaryotic evolutionary tree. Five clades with representative organisms from each clade are shown. (B) Multiple sequence alignments showing parts of N-LIP (top) and C-LIP (bottom) of PAH proteins from various organisms. Assigned colors of the specific residues are based on alignment consensus. The boxes indicate conserved Glycine at the N-LIP and conserved catalytic motif (DXDXT) at the C-LIP. (C) Confocal images of *pah1*Δ yeast cells complemented either with *Trypanosoma PAH* (*TbPAH1*) or with *Arabidopsis PAH* (*AtPAH2*)*.* Both *TbPAH1* and *AtPAH2* rescued the nuclear defect of *pah1*Δ yeast cells. The nucleus is visualized by expression of *PUS-GFP*. Two different transformants per strain were analyzed and the number of cells counted for each one was 200 (*n*=400). The percentage of cells containing a round nucleus is indicated on the right. (D) Rescue of growth defect of *pah1*Δ yeast cells by *TbPAH1* and *AtPAH2* on SD media containing glucose but lacking leucine and uracil at 30°C or 37°C as indicated. Two different transformants per strain were spotted and analyzed.

Prior studies on the role of PAH proteins in the regulation of lipid homeostasis and membrane biogenesis have focused mainly on the Opisthokont and Archaeplastid clades. The cellular function of *PAH* is not yet known in organisms belonging to clades distantly related to Opisthokont, such as the Excavata. Fig. 8A shows an evolutionary tree with representative organisms for each clade. The sequence analysis of *PAH* homologs from organisms belonging to different clades suggest that it is conserved across eukaryotic lineages (Fig. 8B). In this study, we established the role of *PAH1* in regulating lipid homeostasis and membrane biogenesis in *Tetrahymena*, an Alveolate. By complementation of *pah1*Δ yeast cells with *Trypanosoma PAH1* (*TbPAH1*), we further show that conservation appears to extend to another group, the Excavates. *TbPAH1* rescued the growth, respiratory and nuclear defects of *pah1*Δ yeast cells (Fig. 8C,D).

The *Arabidopsis PAH* homolog *AtPAH2* has previously been shown to possess some functions of *S. cerevisiae PAH1*, based on its ability to rescue the slow growth phenotype of *pah1*Δ yeast. However, it was not reported whether the plant homolog also rescues the nuclear envelope defect (Nakamura et al., 2009; Mietkiewska et al., 2011). We used *AtPAH2* to complement the *pah1*Δ yeast strain. In addition to rescuing the growth phenotype, *AtPAH2* mitigated the aberrant nuclear morphology of *pah1*Δ yeast cells, confirming conservation between Opisthokonta and Archaeplastida (Fig. 8C,D). Taken together, these results along with results from earlier reports suggest that the *PAH* phosphatase cascade is functionally conserved across eukaryotic lineages, indicating that it originated before the lineages diverged very early in eukaryotic evolution.

## DISCUSSION

In this study, we have identified two homologs of *LIPIN/PAH* in *T. thermophila.* We report that *TtPAH1* is a phosphatidic acid phosphatase involved in the regulation of lipid droplet biogenesis and ER morphology in *Tetrahymena*. Regulation of lipid homeostasis and membrane biogenesis is fundamental to all eukaryotes, and the presence of a regulation cascade comprising Pah1 and its phosphatase complex Nem1-Spo7 has been shown in yeast (Siniossoglou et al., 1998; Péterfy et al., 2001; Han et al., 2006; Golden et al., 2009; Nakamura et al., 2009). Similar cascades are also reported in plants (Nakamura et al., 2009; Mietkiewska et al., 2011), mammals, (Kim et al., 2007), worms (Golden et al., 2009) and flies (Ugrankar et al., 2011). All studies are restricted to Opisthokonta and Archaeplastida clades. The presence of such a cascade is not reported in the distantly related lower eukaryotic clades such as Alveolata and Excavata. In the present study, we observed that *PAH* from the clades belonging to Excavata and Alveolata functionally replaces yeast *PAH1*. We, therefore, conclude that this phosphatidic acid phosphatase cascade regulating membrane biogenesis and lipid homeostasis is conserved across the eukaryotic evolutionary tree.

Fungi (*S. cerevisiae*), nematodes (*C. elegans*) and insects (*D. melanogaster*) express one *PAH* homolog (Han et al., 2006; Golden et al., 2009; Ugrankar et al., 2011), whereas mammals express three and plants (*Arabidopsis thaliana*) express two homologs (Donkor et al., 2007; Nakamura et al., 2009). The presence of two *PAH* homologs in a lower eukaryote, such as *Tetrahymena*, is unusual since multiple homologs are mainly found in higher organisms. Previous studies have shown that deletion of *PAH* leads to growth and development defects in yeast (Han et al., 2006, 2007; Adeyo et al., 2011), *D. melanogaster* (Ugrankar et al., 2011) and *C. elegans* (Golden et al., 2009). In contrast, loss of *PAH1* in *Tetrahymena* did not result in growth defect. The normal growth and development of Δ*Ttpah1* mutant cells may be attributed to the presence of another homolog, *TtPAH2.*

TtPah1 displays cytoplasmic as well as membrane localization consistent with previously characterized mammalian lipin and yeast Pah1 (Péterfy et al., 2001; Han et al., 2006). Dephosphorylation of Pah1 regulates its subcellular localization and promotes its translocation from the cytoplasm into ER, where it converts PA to DAG (Karanasios et al., 2010). PA phosphatase regulates lipid droplet number by generating its precursor TAG from the substrate PA (Adeyo et al., 2011). The role of *PAH/ LIPIN* in lipid droplet biogenesis or lipid storage has been established in yeast (Adeyo et al., 2011), *Drosophila* (Ugrankar et al., 2011) and *C. elegans* (Golden et al., 2009). By generating the deletion of *TtPAH1 in Tetrahymena*, we demonstrated its role in lipid droplet biogenesis. Overexpression of *TtPAH1* in wild-type cells leads to an increase in lipid droplet number, further corroborating its role in lipid droplet biogenesis.

The role of PAH proteins in maintaining ER structure is well established in yeast and *C. elegans* (Siniossoglou et al., 1998; Campbell et al., 2006; Golden, Liu and Cohen-Fix, 2009). The loss of *PAH* produces a morphological change in many organelles in *Drosophila*, but perturbation of ER morphology was not reported (Ugrankar et al., 2011). Interestingly, deletion of macronuclear copies of *TtPAH1* in *Tetrahymena* alters ER morphology, resulting in an increased proportion of sheet to tubule structure. One possibility for the altered ER morphology could be the change in phospholipid flux arising from the loss of *PAH1*, leading to change in the phospholipid composition of ER. We observed increased intensity of ER-Tracker Red dye in Δ*Ttpah1* cells, indicating higher levels of sulphonyl urea receptor (SUR) of ATP-sensitive K^+^ channel in these cells. The expansion of the ER by deletion of *PAH1* is in general associated with increased expression of membrane proteins that include ER resident proteins in other organisms such as *Schizosacchatamyces pombe*, *C. elegans* and *Yarrowia lipolytica* (Tange, 2002; Golden et al., 2009; Guerfal et al., 2013). Therefore, we speculate that the increased ER membrane synthesis in Δ*Ttpah1* cells concomitantly increases the production of ER-associated proteins and might include ER resident proteins such as SUR. However, this remains to be tested in *Tetrahymena*.

Loss of *PAH* in mammals and *C. elegans* results in a defect in nuclear envelope breakdown (NEBD) without any nuclear expansion (Golden et al., 2009; Gorjánácz and Mattaj, 2009). The regulation of nuclear expansion by *PAH* is restricted to yeast, which could be explained by the presence of the CDP-DAG pathway in yeast and its absence in mammals and *C. elegans* (Bahmanyar et al., 2014; Bahmanyar, 2015). The accumulation of PA due to loss of *PAH1* leads to the excess synthesis of phospholipids PE and PC via the CDP-DAG pathway, resulting in massive nuclear expansion in yeast (Santos-Rosa et al., 2005; Han et al., 2006; Bahmanyar et al., 2014). It is interesting to note that although *Tetrahymena* possesses the CDP-DAG pathway for phospholipid synthesis, nuclear expansion was not visible in Δ*Ttpah1*. Although we have used only *NUP-GFP* as a marker to detect nuclear expansion, it might be useful to test with other nuclear markers as well. However, we believe that *NUP3-GFP* is also a reliable marker since nuclear membrane flares seen in yeast contain assembled nuclear pore structures (Siniossoglou et al., 1998). Further, by staining the nuclear membrane with a lipophilic dye that should stain any membranous structure, we failed to detect any visible flares in both micronucleus and macronucleus of Δ*Ttpah1*. These results suggest that unlike yeast, in which expansion of the nuclear membrane is very prominent, there is no extensive expansion of the nuclear membrane in *Tetrahymena* upon deletion of *TtPAH1*. Nuclear volume in *Tetrahymena* is variable presumably due to differential ploidy level in the MAC (Raikov 1976; Gorovsky 1980; Bodenbender et al., 1992). Therefore, one could speculate a different mechanism that allows plasticity in nuclear expansion to accommodate different nuclear volumes.

The NE is connected with the ER, and changes in ER structure lead to defects in the NE. For example, while overexpression of reticulons and *DP1* inhibits nuclear envelope formation and nuclear expansion, loss of their functions enhances nuclear envelope assembly (Anderson and Hetzer, 2008). In yeast, loss of *PAH1* leads to an overdeveloped ER membrane, which in turn results in nuclear expansion (Siniossoglou et al., 1998; Tange, 2002). As discussed above, loss of *PAH1* in *Tetrahymena* demonstrates a change in ER content and structure. Although the loss of *TtPAH1* increases the ER sheet structure (Fig. 5A), it does not manifest visible defect in the nuclear envelope. It appears that in *Tetrahymena*, unlike in other organisms, ER content and structure are functionally isolated from mechanisms underlying nuclear expansion. However, further studies are required to clearly understand the regulation of nuclear expansion and its relation to ER in *Tetrahymena*.

Mutation of the catalytic motif in TtPah1 leads to loss of function, suggesting that the catalytic activity is necessary for its function. The role of *PAH*, other than catalytic function is identified in other organisms. For example, *PAH* acts as transcriptional co-activator in mammals and as a transcription factor in yeast (Santos-Rosa et al., 2005; Finck et al., 2006; Kim et al., 2013). However, further studies are required to show if *Tetrahymena PAH1* has a direct role in transcription.

*PAH* homolog is functionally conserved (interchangeable) between Opisthokonta and Plantae (Nakamura et al., 2009; Mietkiewska et al., 2011). We have now extended functional conservation of *PAH* to lower eukaryotic lineages such as Alveolata and Excavata. Though there is no report of the presence of such a cascade in Amoebozoa, the *PAH* homolog is present in the genome sequence of *Dictyostelium* (an Amoebozoan). Therefore, it can be concluded that cascade comprising *PAH* for regulation of lipid homeostasis and membrane biogenesis was present in common ancestors before the divergence of lineages, and this cascade remained functionally conserved without allowing change or modification in these functions, since lipid homeostasis and membrane biogenesis regulation is important for the normal growth of all eukaryotes. *PAH*, in addition to lipid homeostasis and membrane biogenesis, has an additional role such as NEBD (in *C. elegans*) (Golden et al., 2009; Bahmanyar et al., 2014) and nuclear expansion (in yeast) (Santos-Rosa et al., 2005), suggesting that the lineage-specific role of *PAH* is adopted after divergence from the common ancestor. However, *PAH* homologs from all the lineages discussed here rescue abnormal nuclear expansion. Therefore, it can be concluded that though all known functions of *PAH* were present before lineage divergence, different lineages have adopted these functions to regulate various cellular processes.

Overall, our results along with results from previous studies as discussed above clearly demonstrate a common regulatory cascade across eukaryotic lineages and may have appeared before the divergence of lineages. Our results also show that unlike other known *PAH* homologs, *TtPAH1* does not regulate nuclear morphology.

## MATERIALS AND METHODS

### Strains and culture conditions

Wild-type CU428.1 and B2086 strains of *T. thermophila* were grown at 30°C in SPP medium (2% proteose peptone, 0.2% dextrose, 0.1% yeast extract, 0.003% ferric EDTA). For conjugation, cells of different mating types were grown to log phase, washed and starved in DMC (0.17 mM sodium citrate, 0.1 mM NaH_2_PO_4_, 0.1 mM Na_2_HPO_4_, 0.65 mM CaCl_2_ and 0.1 mM MgCl_2_) for 16-24 h at 30°C (Orias et al., 2000). For long-term storage, wild-type or knockout cells were starved and frozen in liquid nitrogen in 4% DMSO (Bruns et al., 2000).

### Construction and expression of *TtPAH1-GFP*, *TtPAH1-TAP* and *NUP3-GFP*

To generate the *TtPAH1-GFP* construct, full-length *TtPAH1* was amplified from genomic DNA using specific primers (Table S1). The amplified product was cloned into an entry vector using a pENTR/D-TOPO kit (Invitrogen). This was further cloned into the destination vector pIGF (*Tetrahymena*-specific rDNA-based vector, a gift from Doug Chalker, Washington University, USA) using LR clonase. For expressing *TtPAH1* as TAP-tagged protein, full-length *TtPAH1* was PCR amplified using specific primers with a XhoI restriction site in the forward primer and an Apa1 restriction site in the reverse primer (Table S1), and the amplified product was cloned into *Tetrahymena*-specific vector pVGF (from Meng-Chao Yao, University of Washington, USA) using XhoI and ApaI restriction sites.

*TtPAH1-TAP* and *TtPAH1-GFP* were transformed into wild-type *Tetrahymena* cells using 20 μg of the plasmid by electroporation (Gaertig et al., 1994). Transformants were selected with 100 μg/ml paromomycin sulfate and induced with 1 μg/ml cadmium chloride for 4-5 h to stimulate transcription of the transgene from the *MTT1* promoter. The *NUP3-GFP* in NCVB vector (from Aaron Turkewitz, University of Chicago, USA) was linearized and introduced biolistically into vegetative *Tetrahymena* by particle bombardment, and the transformants were selected using 60 μg/ml blasticidin in the presence of 1 μg/ml cadmium chloride (Rahaman et al., 2008).

### Disruption of *TtPAH1*

5′UTR and 3′UTR of *TtPAH1* were PCR amplified and cloned into the pCRII vector (Invitrogen). To amplify 5′UTR, SacI and EcoRI restriction sites were incorporated in the forward and reverse primer, respectively (Table S1). For amplification of 3′UTR, EcoRI and XhoI restriction sites were included in the forward and reverse primer, respectively (Table S1). Finally, the *NEO3* cassette was introduced between 5′UTR and 3′UTR using EcoRI restriction sites. The resulting knockout construct was linearized by digesting with SacI and XhoI restriction enzymes and introduced biolistically into vegetative *Tetrahymena* by particle bombardment as previously described (Gaertig et al., 1994; Cassidy-Hanley, 2003). The complete replacement of endogenous *TtPAH1* was achieved by growing the transformants in the presence of increasing concentrations of paromomycin sulfate (≤1.2 mg/ml) with 1 µg/ml cadmium chloride.

### Semi-quantitative RT-PCR

Total RNA was isolated from Δ*Ttpah1* cells and wild-type cells using a RNeasy Mini Kit (Qiagen). A QuantiTect Reverse Transcription Kit (Qiagen) was used to synthesize cDNA. PCR reactions were performed with 100 ng cDNA using alpha-tubulin (*ATU1*)- and *TtPAH1*-specific primers (Table S1) in the same reaction for 25-40 cycles.

### Purification of TtPah1-TAP

For purification of TtPah1-TAP, *Tetrahymena* cells harboring *TtPAH1*-pVGF were grown to a density of 3×10^5^ cells/ml. The culture was induced with 1 µg/ml cadmium chloride for 5 h at 30°C, and cells from 300 ml cultures were collected by centrifugation. The cell pellet was resuspended in 10 ml lysis buffer [20 mM Tris-HCl (pH 8.00), 100 mM NaCl, 0.5% NP-40, 10% glycerol] supplemented with a mixture of protease inhibitors (pepstatin, E-64, aprotinin and protease inhibitor cocktail). The lysate was clarified by ultracentrifugation (Optima L100K, 70Ti rotor, Beckman Coulter, Brea, CA, United States) for 1 h at 250,000 ***g***. To minimize proteolysis, all subsequent steps were carried out at 4°C unless mentioned otherwise. Rabbit-IgG agarose slurry (Sigma-Aldrich) pre-equilibrated with wash buffer was added to the clarified lysate and was kept for binding for 2 h. Resin was collected by centrifugation (1 min at 3000 ***g***) and washed with 50 bed volumes of wash buffer [20 mM Tris-HCL (pH 8.00), 2 mM MgCl_2_, 0.2 mM EGTA, 0.1% Tween 20, 10% glycerol, 1 mM DTT, 0.1 mM PMSF]. Resin was incubated with 2 µl TeV protease in 200 µl cleavage buffer [10 mM Tris-HCl (pH 8.00), 0.1 M NaCl, 0.1% Tween 20, 0.5 mM EDTA, 1 mM DTT] for 1.5 h at room temperature, followed by further incubation at 4°C overnight. The eluate after proteolytic cleavage was adjusted to 3 mM CaCl_2_ and mixed with three volumes of calmodulin binding buffer [10 mM Tris-HCl (pH 8.00), 100 mM NaCl, 1 mM Mg acetate, 1 mM imidazole, 2 mM CaCl_2_, 0.1% Tween 20, 10 mM βME]. This was incubated with 100 µl calmodulin resin (GE Healthcare, Buckinghamshire, UK) at 4°C for 1 h. The resin was recovered by centrifugation and washed with calmodulin binding buffer. Protein was eluted with calmodulin elution buffer [10 mM Tris-HCl (pH 8.00), 100 mM NaCl, and 1 mM Mg acetate, 1 mM imidazole, 10 mM EGTA, 0.1% Tween 20, 10 mM 2-mercaptoethanol] (Witkin and Collins, 2004). Eluted fractions were loaded on 10% SDS polyacrylamide gel, and the protein was detected by silver staining.

### Growth analysis

*TtPAH1* knockout cells and wild-type cells were grown in triplicate. When the cell number reached 1×10^5^/ml, cells were counted using a hemocytometer at 2 h intervals after fixation with formalin. The averaged cell density was plotted against time.

### Isolation of nuclei

*Tetrahymena* cells (50 ml, 5×10^5^ cells/ml) were centrifuged (5 min at 1100 ***g***) at 4°C and cell pellets were washed with pre-chilled Solution A (sucrose 0.1 M, gum arabic 4% v/v, MgCl_2_ 0.0015 M, Spermidine Hydrochloride 0.01% v/v) and resuspended in pre-chilled Solution B (sucrose 0.1 M, gum arabic 4% v/v, MgCl_2_ 0.0015 M, Spermidine Hydrochloride 0.01% v/v, octanol 24 mM). The suspension was shaken vigorously for 5 min followed by centrifugation (Allen, 2000). The nuclear pellet was resuspended in Buffer A and imaged by fluorescence microscope after staining with DAPI.

### Staining and microscopy

For staining lipid droplets, *Tetrahymena* cells were pelleted down by centrifugation (1100 ***g*** for 2 min) at room temperature, washed with DMC and fixed with 4% paraformaldehyde. Fixed cells were washed with 10 mM HEPES and resuspended in the freshly prepared Oil Red O solution. Cells were tapped briefly and incubated in the dark in a nutating mixer at room temperature for 10 min. Stained cells were washed three times with 10 mM HEPES and resuspended in 10 mM HEPES before imaging in a confocal microscope (Binns et al., 2006).

For ER staining, *Tetrahymena* cells were grown to a density of 3-4×10^5^ cells/ml, and 0.5 µM ER-Tracker Red dye (Invitrogen) was added to the culture and incubated for 60 min before fixing with 4% paraformaldehyde (50 mM HEPES, pH 7.5). To rule out any effect of differential pressure (during placing coverslips) on ER morphology in different samples, we imaged both wild-type cells and knockout cells simultaneously.

For Oil Red O staining images were taken at 543 nm excitation/619 nm emissions and for ER-Tracker Red images were taken at 587 nm excitation/615 nm emissions. Then, 3-5 µl of cells were mounted on glass slides, covered with cover glasses, sealed with nail polish and imaged with a LSM780 confocal microscope (Zeiss, Oberkochen, Germany).

For staining *Tetrahymena* nucleus, it was incubated with 5 μg/ml DHCC and 0.5 μg/ml DAPI for 10 min in dark, washed three times with Solution A and resuspended in the same solution before imaging in an Eclipse Ti fluorescence microscope (Nikon, Tokyo, Japan).

To quantitate ER content, the stacked images of ER-Tracker Red-stained cells were analyzed by ImageJ (https://imagej.nih.gov/ij/) after sum intensity projection. The mean intensity values were plotted for both wild-type (*n*=34) and Δ*Ttpah1* (*n*=32) cells using box plot.

### Gene synthesis

The coding region of *TtPAH1* was commercially synthesized (Eurofins, Louisville, KY, USA) after codon optimization and obtained in the pUC57 vector. This commercially synthesized gene was used for expression in bacteria and complementation assays in yeast.

### Yeast culture conditions

Yeast cells were grown either in yeast extract peptone dextrose (YPD) medium or synthetic complete dextrose (SD) media containing 2% glucose with appropriate amino acids (Sherman, 2002). For growth analysis, yeast cells were grown in SD medium lacking leucine and uracil to early logarithmic phase, serially diluted (10-fold) and 5 µl of each dilution was spotted onto the solid SD medium lacking leucine and uracil and incubated at either 30°C or 37°C for 2-4 days. To check respiratory deficiency, glycerol (2%) in place of dextrose was used as the carbon source.

### Site-directed mutagenesis

Point mutations (D666,668E) at the corresponding sites of the *TtPAH1* coding region in *YCplac111-PAH1* fusion construct were introduced using a Quik Change Site-Directed Mutagenesis protocol (Stratagene), and the mutations were confirmed by DNA sequencing.

### Yeast complementation assay

The full-length coding sequence of *TtPAH1* (*T. thermophila PAH1*), *AtPAH2* (*A. thaliana PAH2*) and *TbPAH1* (*Trypanosoma brucei PAH1*) were amplified using specific primers and cloned into YCplac111 (LEU) using SalI/ BamHI restriction sites. To assess nuclear membrane morphology and growth rescue, *pah1*Δ yeast cells (RS453 smp2Δ: ade2his3leu2trp1ura3 smp2::TRP1 were transformed with either *TtPAH1* or *TtPAH1_mut_* or *AtPAH2* or *TbPAH1* along with *PUS-GFP* by standard lithium acetate protocol (Gietz and Woods, 2001). Transformants were screened on solid SD medium lacking uracil and leucine. The transformants were grown in the same media at 30°C to early log phase and analyzed by confocal microscopy. The results from three independent experiments were used for analysis of nuclear morphology.

### Analysis of gene expression

Gene expression was analyzed by RT-PCR by isolating total RNA from cells grown in SD media containing adenine and histidine. The isolated RNA was used to synthesize single-stranded cDNA using Superscript II reverse transcriptase (Invitrogen). For quantitative analysis, RT-PCR was performed using the SYBR Green qPCR (Roche) in a 7500 Real-Time PCR System (Applied Biosystems, Foster City, CA, USA) following the manufacturer's instructions. All primer sequences used are listed in Table S1. The relative expression level was calculated using the comparative Ct method after normalizing to SEC 63 as a control gene.

### Phosphatase assay

Phosphatidic acid phosphatase activity was measured by following the release of water-soluble Pi from chloroform-soluble PA. The standard reaction contained 50 mM Tris-HCl buffer (pH 7.5), 1 mM MgCl_2_, 10 mM Triton X-100, 10 mM 2-mercaptoethanol and 1 mM phosphatidic acid in a total volume of 100 μl. Reactions were initiated by the addition of recombinant proteins and carried out in triplicate at 30°C for 20 min. The reaction was terminated by adding 500 µl of 0.1 M HCl in methanol and 1 ml chloroform. To that mixture, 1 ml of water was added for phase separation, and one volume of upper phase was mixed with two volumes of Biomol Green to develop color. The absorbance was measured at 620 nm, and the amount of phosphate produced was quantified using a standard curve (Han and Carman, 2010).

### Sequence analysis

Sequences of *Tetrahymena PAH* homologs (TTHERM_00189270 and TTHERM_00215970) were retrieved from the Tetrahymena Genome Database and domains were predicted with Interpro protein sequence analysis and classification tool (EMBL-EBI). Multiple sequence alignment was performed with PRALINE. Percent identity matrix was calculated using Clustal2.1. The sequences of PAH used in this study were S000004775 for *S.* *cerevisiae*, NM_001203528.1 for *A.* *thaliana* (*AtPAH2*), XM_841075 for *Trypanosoma brucei*, FBgn0263593 for *D.* *melanogaster*, BC030537.1 for *Homo sapiens* and *DDB_G0271730* for *Dictyostelium discoideum.*

## Supplementary Material

Supplementary information
