# Supplementary material for: An evolutionarily conserved phosphatidate phosphatase maintains lipid droplet number and endoplasmic reticulum morphology but not nuclear morphology
Source: Biol Open. 2017 Sep 27;6(11):1629–43. doi: 10.1242/bio.028233 (PMC5703613; doi:10.1242/bio.028233)
Supplement: Supplementary information [file biolopen-6-028233-s1.pdf]

Biology Open (2017): doi:10.1242/bio.028233: Supplementary information

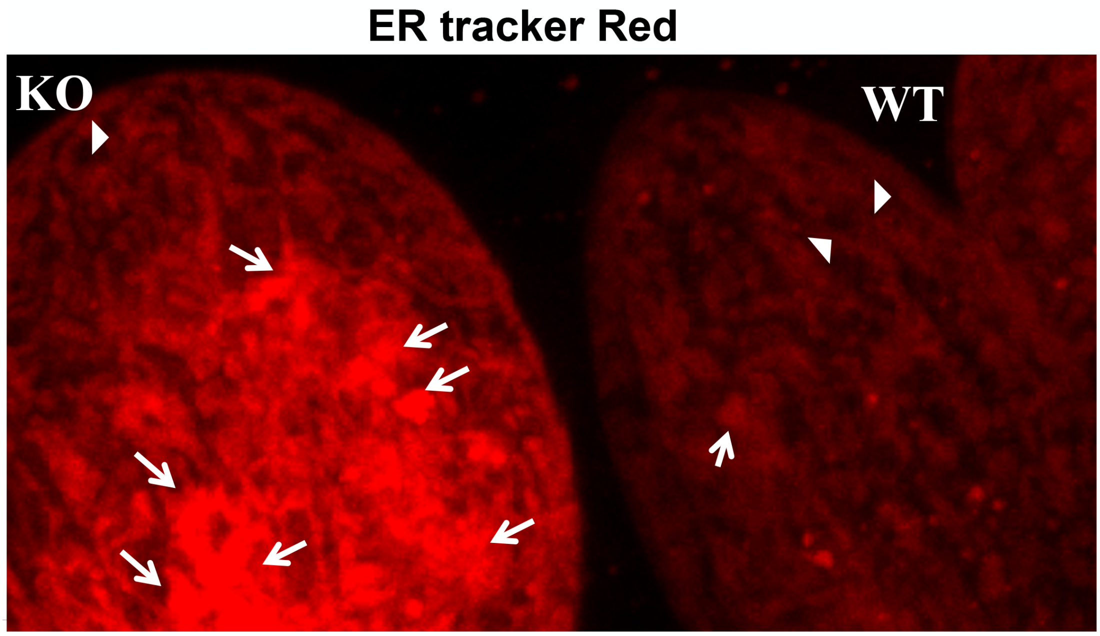

Figure S1: Confocal stack of Δ*Ttpah1* (KO) and wild-type (WT) cells stained with ER-tracker Red. Arrow indicates ER sheet and arrowhead indicates ER tubule structures.

Table S1: Oligonucletides used in this study. (The underlined sequences represent sequences of restriction site added to the oligonucleotides.)

**Oligo Name**	**Sequence**
TtPAH1-GFP FP	CACCATGCATCACCATCACCATCACACTACTAGCAGTATGAGTGTTTTAA
TtPAH1-GFP RP	TCATTCGCTTAATAGTTAGTTAATATCTT
TtPAH1-TAP FP	GCCTCGAGACT AGCAGTATGAGTGTTTTAA
TtPAH1-TAP RP	GCGGGCCCTCATTCGCTTAATAGTTAGTTAATATCTT3
5'UTR FP	GAGCTCGTGAATAGTAGTAATCTTAA
5'UTR RP	GAATTCCTTAATCAGAAATTATGATAATATATCT
3'UTR FP	GAATTCTTTCCTCCCATCAAGCAG
3'UTR RP	CTCGAGTTCTATTTATATTTTTTGTTAA
TtPAH1 RT FP	GCTATTGGGCAATCGGAGTA
TtPAH1 RT RP	TCCAAATCCTGCATAATAGACG
Alpha tubulin RT FP	CCTCCCCCTAAGTCTCAACC
Alpha tubulin RT RP	CGAAGGCAGAGTTGGTGATT
INO1 FP	CATGGTTAGCCCAAACGACT

Biology Open • Supplementary information

INO1 RP	CGTGGTTACGTTGCCTTTTT
INO2 FP	TTCCAGCCAATATCGAGGAC
INO2 RP	AGTGCTTCATTTGCGCTTCT
OPI3 FP	ACATGGTGTACGAGTCTGCA
OPI3 RP	CATGGGGTTGTTGGAAACGT
SEC-63 FP	TCCTTGTCCCAGGTGAGAAC
SEC-63 RP	ATGGGACGAGTGGCTGTTTA
TtPAH1mut FP	ATGTCCAGATTGTAATTTCAGAGATAGAGGGAACAATCACCAAATCTGATG
TtPAH1mut RP	CATCAGATTTGGTGATTGTTCCCTCTATCTCTGAAATTACAATCTGGACAT
TtPAH1 FP	GCGTCGACATGAGTGTTTTAAAAAAACTACAG
TtPAH1 RP	GCGGATCCTCATTCGCTTAATAGCTGGTTAAT
AtPAH2 FP	GCGTCGACCTCGAGATGAATGCCGTCGGTAGGATC
AtPAH2 RP	GCGGATCCGTTTAAACTCACATAAGCGATGGAGGAGG
TbPAH1 FP	GCCTCGAGATGATATCTGGTTTTGCAGATTTC
TbPAH1 RP	GCGGATCCTCACACAGTGTCACCTTGTTG

Biology Open • Supplementary information
